# Fluorescence-Guided Surgery Using 5-Aminolevulinic Acid/Protoporphyrin IX in Brain Metastases

**DOI:** 10.1227/neuprac.0000000000000121

**Published:** 2024-11-19

**Authors:** Anthony Price, Joshua D. Bernstock, Nina Truong, Kyle Wu, John Y. K. Lee, Isaac J. Tucker, Florian Gessler, Salvatore DeSena, Gregory Friedman, Pablo A. Valdes

**Affiliations:** * John Sealy School of Medicine, The University of Texas Medical Branch at Galveston, Galveston, Texas, USA;; ‡ Department of Neurosurgery, The University of Texas Medical Branch at Galveston, Galveston, Texas, USA;; § Department of Neurosurgery, Harvard Medical School, Brigham and Women's Hospital, Boston, Massachusetts, USA;; ‖ David H. Koch Institute for Integrative Cancer Research, Massachusetts Institute of Technology, Cambridge, Massachusetts, USA;; ¶ Department of Neurosurgery, Ohio State University, Columbus, Ohio, USA;; # Department of Neurosurgery, University of Pennsylvania, Philadelphia, Pennsylvania, USA;; ** Institute for Molecular Bioscience, The University of Queensland, Brisbane, Queensland, Australia;; †† Department of Neurosurgery, University of Rostock, Rostock, Germany;; ‡‡ NX Development Corp, Lexington, Kentucky, USA;; §§ Department of Neurosurgery, The University of Texas MD Anderson Cancer Center, Houston, Texas, USA;; ‖‖ Department of Neurobiology, The University of Texas Medical Branch at Galveston, Galveston, Texas, USA;; ¶¶ Department of Electrical and Computer Engineering, Rice University, Houston, Texas, USA

**Keywords:** 5-Aminolevulinic acid (5-ALA), Brain metastases, Fluorescence-guided surgery (FGS), Protoporphyrin IX (PpIX)

## Abstract

**BACKGROUND AND OBJECTIVES:**

The purpose of this systematic review was to provide a comprehensive overview of the available literature on 5-aminolevulinic acid (5-ALA)–induced protoporphyrin IX (PpIX) fluorescence-guided surgery (FGS) for the resection of brain metastases (BMs).

**METHODS:**

A comprehensive search of the PubMed database for literature on 5-ALA use in BMs surgery was performed. For inclusion, BMs studies had to have data on the observed intraoperative fluorescence available. Additional data categories included the number of metastatic tumors, 5-ALA dosage and timing, the imaging system (eg, microscope) used, imaging wavelength(s), fluorescence grading (“simple” and “detailed”), fluorescence consistency (heterogeneous vs homogeneous), intracranial tumor location, metastatic primary tumor location, and extent of resection, among others.

**RESULTS:**

Twenty-three articles published between 2007 and 2022 met the inclusion criteria. These studies comprised 1709 total patients; 870 metastatic samples were collected from 855 patients with 377 (43.3%) fluorescence-negative and 493 (56.7%) fluorescence-positive samples. The pooled overall prevalence of fluorescence-positive metastatic lesions was 66% (95% CI 55%-75%; I^2^ = 85%, *P* < .01). The fluorescence grading was as follows: (a) simple fluorescence (n = 599): 295 (49.3%) fluorescence-negative and 304 (50.8%) fluorescence-positive samples and (b) detailed fluorescence (n = 271): 82 (30.3%) no fluorescence, 107 (39.5%) weak fluorescence, and 82 (30.3%) strong fluorescence. A total of 764 lesions had primary tumor site data available: 702 lesions had fluorescence data with 384 (54.7%) fluorescence-positive samples.

**CONCLUSION:**

FGS using 5-ALA/PpIX in BMs demonstrates varying benefits as an adjunct for maximizing the extent of resection. Thus, preoperative knowledge of the primary tumors' origin may inform surgeons regarding the potential utility of 5-ALA/PpIX for FGS management of BMs.

**ABBREVIATIONS:**
5-ALA: 5-aminolevulinic acid
BMs: brain metastases
CNS: central nervous system
EOR: extent of resection
FGS: fluorescence-guided surgery
HGG: high-grade glioma.

Brain metastases (BMs) are the most common central nervous system (CNS) tumors and are 10 times more frequent than primary malignant brain tumors, occurring in over 25% of all patients with cancer.^1,2^ Surgery has been shown to influence local recurrence rates, overall survival, and quality of life.^3,4^ Therefore, the goal of neuro-oncological surgery is to maximize the extent of resection (EOR) while preserving adjacent functional brain.^5^ BMs have recently been shown to have an infiltrating margin beyond the typical glial pseudocapsule, which significantly affects survival.^6^ Although gross total resection may be observed on postoperative clinical imaging studies, undetected and infiltrating metastatic cells may account for ∼60% of local recurrences.^7,8^ Accordingly, recent studies have shown that supramarginal resection of both gross and microscopic infiltrating disease led to >50% lower local recurrence rates and increased 2-year overall survival in patients with BMs (27% vs 4%).^9^

Fluorescence-guided surgery (FGS) seeks to improve the safety and efficacy of surgery through direct visual identification of tumor tissues that require removal and/or structures that must be preserved to optimize postoperative function (eg, vessels, nerves). FGS provides an opportunity for real-time visualization of tumor tissue using a specific fluorescent agent (eg, 5-aminolevulinic acid/protoporphyrin IX [5-ALA/PpIX]). Most of the literature reports a high level of fluorescence in high-grade glioma (HGG) tissue, where 5-ALA/PpIX has become an adjunct in the standard of care for the resection of HGG.^10-15^ However, the development of targeted fluorophores for labeling BMs has received little attention, with no Food and Drug Administration (FDA)-approved agents for use in BMs, despite the clinical benefits of maximal safe resection of BMs.^16,17^ Given the success of 5-ALA-PpIX in HGG, ongoing studies are currently underway evaluating its diagnostic efficacy and safety in other intracranial and extracranial pathologies.^18-23^

The aim of this systematic review was to analyze the literature on 5-ALA/PpIX use in BMs and provide an evidence-based analysis of 5-ALA/PpIX FGS for metastatic CNS tumors. Given the success of FGS for the resection of HGG and the importance of maximally safe resection of BMs, a comprehensive review of this literature is necessary to inform neurosurgeons regarding the perioperative surgical option using 5-ALA/PpIX and to inform future clinical and research directions to advance the field of FGS beyond HGG into additional neuro-oncologic applications. Given the significant burden of CNS disease BMs represent, the greatest surgical impact for FGS may ultimately be observed in BMs.^24^

## METHODS

### Study Selection Criteria

**FIGURE 1.**
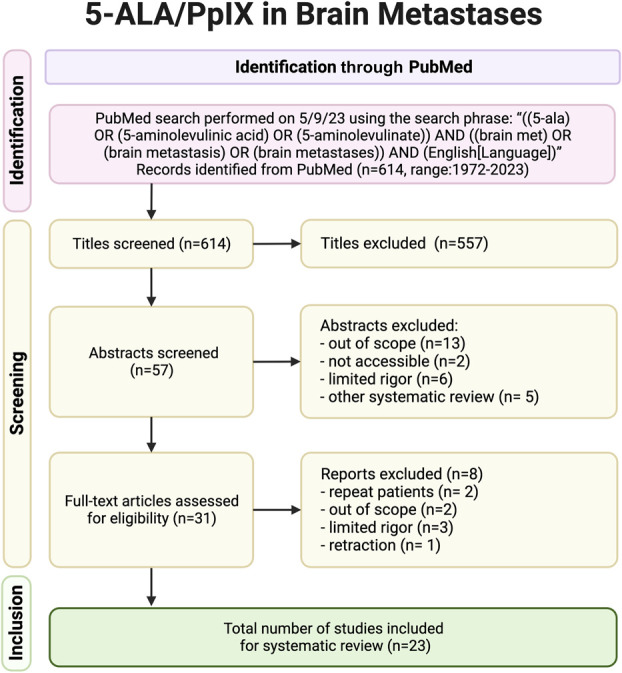
5-ALA/PpIX in brain metastases Preferred Reporting Items for Systematic Reviews and Meta-Analyses flow diagram: n = number, scope (metastases with simple fluorescence data, denoted as either “yes” or “no” visible fluorescence data available at a minimum to be considered), limited rigor (1 abstract literature review, 1 abstract focusing on frozen sections, 1 abstract focusing on methylation status, 3 case reports). 5-ALA, 5-aminolevulinic acid; PpIX, induced protoporphyrin IX.

In this review article, we discuss the clinical experience with 5-ALA in metastatic brain tumors. Although 5-ALA is FDA approved for use as an intraoperative optical imaging agent in patients with suspected high-grade gliomas, it is not approved by the FDA for use with brain metastasis. A search of the PubMed database for literature on 5-ALA use in BMs was performed on May 9th, 2023. All abstracts were screened by 2 reviewers, “A. P.” and “P. A. V.” A diagram of the screening process using the Preferred Reporting Items for Systematic Reviews and Meta-Analyses guidelines is shown in Figure 1. Any final articles were evaluated in full for any primary source results of patients undergoing 5-ALA–assisted resection of BMs. Metastases had to have simple fluorescence data, denoted as either “yes” or “no” visible fluorescence at a minimum to be considered for inclusion within this review; articles that did not meet this criterion were excluded. As 5-ALA has been mostly used in gliomas, studies focused partially or primarily on glioma were included if they included metastatic patient data. The nonmetastatic patients in these metastatic studies such as low-grade gliomas, high-grade gliomas, and meningiomas are reported separately (see **Supplemental Digital Content 1** [http://links.lww.com/NS9/A33**]**). This study was not registered in the International Prospective Register of Systematic Reviews (PROSPERO), as the registry only accepts reviews before any data collection has started. As the data were collected from prior studies without protected health information, no protocol was needed from the first and senior author's institution. Full statistics including the Egger regression addressing any potential bias found are addressed below.

### Data Evaluation

A total of 23 articles that met inclusion criteria were assessed for comparable data categories. The following data categories were included in the final analyses: study design (prospective vs retrospective), number of metastatic tumors, age, sex, 5-ALA dosage and timing, the imaging system (eg, microscope) used, imaging wavelength(s) used, fluorescence grading (ie, “simple” and “detailed”), fluorescence consistency (ie, heterogeneous vs homogeneous), intracranial tumor location, metastatic primary tumor location, and EOR. Multiple other data categories with no data or not enough data recorded for meaningful comparisons were excluded (eg, data available in ≤5 studies) but are included in the **Supplemental Digital Content 2** (http://links.lww.com/NS9/A34).

We have classified the qualitative descriptions of the visual fluorescence grading reported by surgeons as “simple or detailed.” Simple was reported as “fluorescence-negative” or “fluorescence-positive” in 19 studies. Detailed was reported as “negative,” “weak/vague,” or “strong” in 4 articles. Belloch et al^25^ reported their visual fluorescence grading from 1 to 5 (5 being maximum) in 3 metastases. As Belloch et al^25^ did not specify grade 1 intensity lacked fluorescence, the 2 *grade 1* metastases were considered as “weak” fluorescence for this analysis. The single instance of Belloch *grade 4* intensity metastasis was considered a “strong” fluorescence. The Dartmouth group reported their visual fluorescence grading from 0 (none) to 4 (high) in 4 metastases.^26^ In this study, 3 metastases samples with grade 1 fluorescence were considered “weak” while the singular sample with grade 2 fluorescence was considered “strong” using our detailed classification system.^26^ Fluorescence assortment for this analysis was assessed using the intraoperative visible fluorescence when available. Here we report on the visible fluorescence as observed through the surgical microscope, which has been described by our group as “qualitative fluorescence”. The only work noted with “quantitative” fluorescence derived using spectroscopic measurements with handheld probes was by Valdés et al.^19,27^ The nonmetastatic tumors in the metastatic studies included here were also organized into “simple fluorescence” fluorescence-negative and fluorescence-positive (**Supplemental Digital Content 3** [http://links.lww.com/NS9/A35]).

The intracranial locations of tumors were described as either supratentorial (ie, frontal, temporal, etc.) or infratentorial (ie, cerebellar or unspecified) when available. The primary tumor types extracted and evaluated were the lung, breast, gastrointestinal/colorectal, urogenital, melanoma, renal cell, squamous cell carcinoma (skin), and “other” primary types. Lung cancer subgroups were also extracted. The study by Belloch et al^25^ was the only article to quantify values for their EOR, so a similar system was adapted here. The EOR based on T1-weighted postcontrast MRI for this analysis was described simply as incomplete or complete as specific percentages were unavailable.

### Statistics

Analyses and graphs except the bias statistics were completed using GraphPad Prism 10. Two image schematic figures included in this article were created using BioRender.com templates. χ^2^ tests with Yates continuity correction were run between the categorical variables. A proportional meta-analysis was performed to calculate the pooled prevalence estimates using a random-effects model with the DerSimonian and Laird approach. Heterogeneity was evaluated through the I^2^ statistic. Pooled data are presented in forest plots with 95% CIs. The forest plot, funnel plot, and Egger regression were completed using RStudio version 4.3.1 using the “meta” package version 6.5-0.^28^

### Data availability

All data used are included in the article or the supplemental files. Any additional data or clarifications on data are encouraged by email with the corresponding author on request.

## RESULTS

### Study Selection

The initial PubMed database search returned 614 results published between the years 1972 and 2023 (Figure 1; **Supplemental Digital Content 2** [http://links.lww.com/NS9/A34]). The second phase of screening focusing on the relevance of 57 resulted in the exclusion of an additional 26 articles: 13 were deemed to be out of scope, 2 were not accessible, 6 for limited rigor (1 literature review, 1 focusing on frozen sections, 1 focusing on methylation status, 3 case reports), and 5 systematic reviews. The 31 remaining articles were evaluated in full for results related to patients undergoing 5-ALA/PpIX FGS of BMs. After a full review of the remaining articles, a further 8 studies were excluded based on the following: 2 for repeat patients (Kamp et al, 2012,^29^ 2016^30^), 2 out of scope (metastases that turned out the be gliomas^31^ and old metastases as controls^32^) 3 for limited rigor (only 1 metastasis with fluorescence, glioblastoma recurrences defined as metastases, or no fluorescence data),^33-35^ and 1 for retraction. As such, a total of 23 manuscripts, published between 2007 and 2022, were ultimately included in the analyses having met the inclusion criterion (Table 1).

**TABLE 1.** Characteristics of Evaluated Studies

5-ALA fluorescence by study											
Study (n = 23)	Design	Dose & timing	No. Pts	Avg. Age (range)	Sex		Metastasis	Fluorescence		Microscope & working distance	Wavelength
					Male	Female		Fluor-neg	Fluor-pos		
Utsuki et al,^36^ 2007	Prospective	1 g 4-6 h before	6	∅	∅	∅	6	2 (33.3)	4 (66.6)	∅	405 nm
Utsuki et al,^37^ 2007	Prospective	1 g 4-6 h before	11	∅	∅	∅	11	2 (18.2)	9 (81.8)	IX70; Olympus	405 nm
Valdés et al,^27^ 2011	Prospective	20 mg/kg 3 h prior	14	∅	∅	∅	3	3 (100)	0 (0)	OPMI Pentero, Carl Zeiss	400 nm
Suero Molina et al,^38^ 2013	Prospective	20 mg/kg 4 h prior	26	51.9, (29-77)	6	1	7	0 (0)	7 (100)	OPMI Pentero, Carl Zeiss	375-440 nm
Belloch et al,^25^ 2014	Prospective	20 mg/kg 3 h prior	23	56 med, (20-79)	13	8	3	0 (0)	3 (100)	wide field, target distance of 200 mm; HD-XOscope, HD-XOSCOPE, Karl Storz	440 nm
Coburger et al,^39^ 2014	Prospective	20 mg/kg 4 h prior	42	59, (32-82)	∅	∅	11	3 (27.3)	8 (72.7)	OPMI Pentero, Carl Zeiss	405 nm
Marbacher et al,^40^ 2014	Retrospective	20 mg/kg 3-5 h prior	326	59.1 (18-88), 60 med	∅	∅	65	31 (47.7)	34 (52.3)	OPMI Pentero, Carl Zeiss	400-410 nm
Yagi et al,^41^ 2017	Retrospective	20 mg/kg 2 h prior	16	54	11	5	16	11 (68.8)	5 (31.2)	OPMI Pentero, Carl Zeiss	greatest at 405 nm
Choo et al,^42^ 2018	Retrospective	20 mg/kg 3 h prior	7	47.5	0	2	2	0 (0)	2 (100)	2.7-mm 0° rigid EndoArm HD; Olympus	∅
Roberts et al,^26^ 2018	Prospective	20 mg/kg 3 h prior	4	∅	∅	∅	4	0 (0)	4 (100)	OPMI Pentero, Carl Zeiss	620-640 nm
Barbagallo et al,^34^ 2019	Prospective	∅	52	57.67	∅	∅	8	2 (33.3)	6 (66.6)	∅	∅
Goryaynov et al,^43^ 2019	Retrospective	20 mg/kg 2 h prior	493	∅	∅	∅	72	11 (15.3)	61 (84.7)	OPMI Pentero, Carl Zeiss	∅
Ji et al,^44^ 2019	Retrospective	20 mg/kg 3-4 h prior	23	∅	∅	∅	23	3 (13.0)	20 (87.0)	OPMI Pentero, Carl Zeiss; Leica M720 OH5	∅
Kamp et al,^45^ 2019	Retrospective	20 mg/kg 3 h prior	218	62 med, (26-87)	103	115	218	156 (71.6)	62 (28.4)	OPMI Pentero, Carl Zeiss; Leica M530 OH6	405 nm
Knipps et al,^46^ 2019	Prospective	20 mg/kg 3 h prior	29	63 med, (37-81)	15	14	29	16 (55.2)	13 (44.8)	OPMI Pentero, Carl Zeiss; Leica M530 OH6	400 nm
Marhold et al,^47^ 2019	Prospective	20 mg/kg 3 h prior	154	61, (27-84)	74	80	157	53 (33.8)	104 (66.2)	NC4 or Pentero; Carl Zeiss	400 nm
Schatlo et al,^48^ 2019	Prospective	20 mg/kg 3 h prior	27	62	13	14	27	4 (14.8)	23 (85.2)	∅	∅
Erkkilä et al,^49^ 2020	Prospective	20 mg/kg 3 h prior	21	∅	∅	∅	4	0 (0)	4 (100)	Custom System	405 nm
Hussein et al,^50^ 2020	Retrospective	20 mg/kg 3 h prior	56	24 pt >65 yo, 32 pt <65 yo	29	27	56	17 (30.4)	39 (69.6)	∅	∅
Mercea et al,^51^ 2021	Prospective	20 mg/kg 3 h prior	55	62 med, (27-82)	27	28	58	22 (37.9)	36 (62.1)	NC4 or Pentero, Carl Zeiss	∅
Reichert et al,^52^ 2021	Prospective	∅	28	∅	13	15	35	16 (45.7)	19 (54.3)	OPMI VISU 200, Carl Zeiss	405 nm
Bettag et al,^53^ 2022	Retrospective	20 mg/kg 4 h prior	26	21pt > 50 yo	16	10	26	4 (15.4)	22 (84.6)	OPMI Pentero, Carl Zeiss	∅
Marhold et al,^54^ 2022	Retrospective	20 mg/kg 3 h prior	27	63.4 (32-81), 67 med	14	13	29	21 (72.4)	8 (27.6)	NC4, Pentero, Carl Zeiss	∅

5-aminolevulinic acid (5-ALA); Fluor = fluorescence.

∅= data not available from source; Avg. Age (Range) reported as: XX = mean; XXmed = median; (XX-XX) = age range; Fluorescence reported as: XX = number (percentage %).

Authors, study designs (prospective/retrospective), doses and timing of 5-ALA administration, patient details, and micro included in this review.

### Data Evaluation and Extraction

Data on the qualitative^25-27,34,36-54^ visual descriptions of the observed fluorescence (ie, fluorescence positive/fluorescence negative; negative/weak/strong fluorescence) were extracted. We found no consistent age descriptions among studies (data reported as mean, median, and/or age range), so all reported means, medians, and age ranges were extracted from the available studies. The OPMI Pentero microscope (Carl Zeiss) was the most used microscope reported in 14 studies (Table 1). EOR was described in 303 cases (35.4%; n = 303/855) of the population. There were 90 (29.7%; n = 90/303) incomplete and 213 (70.3%, n = 213/303) complete resections (Table 2).

**TABLE 2.** Fluorescence Data on Patients With Brain Metastases

Fluorescence data on brain metastases patients				
	**No.**			
Patients with metastasis	855			
Metastasis samples	870			
**Metastatic fluorescence samples (n = 870)**	**Negative (%)**	**Positive (%)**	**Weak (%)**	**Strong (%)**
Total fluorescence	377 (43.3)	493 (56.7)		
Simple fluorescence	295 (49.3)	304 (50.8)		
Detailed fluorescence	82 (30.3)	189 (69.7)	107 (39.5)	82 (30.3)
**Fluorescence consistency (n = 143)**	**No. (%)**			
Consistency	143 (16.4)			
Heterogeneous	116 (81.1)			
Homogeneous	27 (18.9)			
N/A	727 (83.6)			
**Resection extent (n = 303)**	**No. (%)**			
Resection extent	303 (35.4)			
Incomplete	90 (29.7)			
Complete	213 (70.3)			
N/A	552 (64.6)			
**Location of tumor (n = 411)**	**No. (%)**			
Location reported	411			
Supratentorial	**332 (80.8)**			
Frontal	96 (23.4)			
Temporal	60 (14.6)			
Parietal	37 (9.0)			
Occipital	41 (10.0)			
Central region	8 (2.0)			
Trigonal	5 (1.2)			
Unclassified	85 (20.7)			
Infratentorial	**77 (18.7)**			
Cerebellar	51 (12.4)			
Unclassified	26 (6.3)			
N/A	**2 (0.5)**			

No. (%), number of (percentage).

Fluorescence data of metastatic samples, with fluorescence reported as simple (positive/negative) or detailed (negative/weak/strong) fluorescence. Fluorescence consistency reported as heterogeneous or homogenous. Extent of resection reported as incomplete, complete, or not specified shown as N/A. Location of tumor reported as supratentorial [frontal, temporal, parietal, occipital, central, trigonal, or unclassified], infratentorial [cerebellar or unclassified], or not specified shown as N/A. Bolded tumor locations indcate the percentage of the total tumor location.

### Fluorescence Data

**FIGURE 2.**
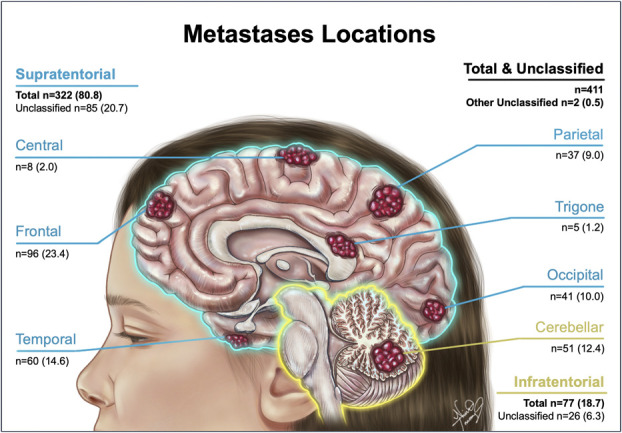
Brain metastases locations. Tumors are displayed by their supratentorial or infratentorial locations. n = number (percentage %). *© 2024 Nina Truong All rights reserved*.

Intracranial location information was available for 411 of 870 (47.2%) tumors (Figure 2). Most tumors were supratentorial (80.8%), compared with a smaller proportion that were infratentorial (18.7%), or reported as unclassified tumor locations (0.5%) (Table 2, Figure 2). The most common supratentorial location was frontal (23.4%), followed by temporal (14.6%) and occipital (10.0%). Cerebellar lesions consisted of 12.4% of tumors.

There was a total of 1709 patients in the studies that fit inclusion criteria (**Supplemental Digital Content 4** [http://links.lww.com/NS9/A36]), of which 855 patients (50.0%) had metastatic tumors and 854 patients (50.0%) had non-metastatic tumors (**Supplemental Digital Content 1** [http://links.lww.com/NS9/A33]). In the patient population with metastatic disease, there were 870 (49.3%) metastatic lesions compared with 896 (50.7%) nonmetastatic lesions. There were almost equal numbers of male and female patients (39.1% male and 38.8% female), and 22.1% did not have patient sex reported (**Supplemental Digital Content 4** [http://links.lww.com/NS9/A36]). Visual fluorescence grading was reported in 870 metastatic lesions, 56.7% were fluorescence-positive and 43.3% were fluorescence-negative. The 870 metastatic lesions were further broken down into samples described by simple fluorescence (positive/negative) and samples described by detailed fluorescence (negative, weak, strong). There were 49.3% fluorescence-negative and 50.8% fluorescence-positive lesions described by simple fluorescence. The detailed fluorescence grading had more fluorescence-positive lesions than the simple fluorescence grading (69.7% vs 50.8% fluorescence-positive lesions), indicating more positives were reported when the grading scale was more discretized: 30.3% fluorescence-negative, 39.5% fluorescence-weak, and 30.3% fluorescence-strong metastatic lesions (**Supplemental Digital Content 5** [http://links.lww.com/NS9/A37]).

There were 143 samples with data on fluorescence consistency with most fluorescence described as heterogeneous in 81.1% and only homogeneous in 18.9% of lesions. A total of 764 lesions had tumor primary site data available (**Supplemental Digital Content 5** [http://links.lww.com/NS9/A37]), of which 702 lesions had fluorescence data with 54.7% fluorescence-positives (Table 3). The highest fluorescence positivity was seen in renal cell carcinoma metastases (66.7%), breast metastases (63.3%), and lung cancer metastases (57.8%). Of the lung primary site metastases, small cell lung cancer metastases had the highest fluorescence-positivity (62.5%), followed by 50.5% fluorescence-positive nonsquamous cell lung cancer metastases and 50.0% fluorescence-positive squamous cell lung cancer metastases. Melanoma metastases (40.2%), urogenital metastases (38.5%), and squamous cell carcinomas of skin metastases (22.2%) were the least likely to display the fluorescence positivity.

**TABLE 3.** Fluorescence Data Based on Sex, Primary Tumor Site, and Histology

Fluorescence data based on sex, primary tumor site, and histology						
Patients with metastasis	855					
**Sex**	**No. (%)**					
Male	334 (39.1)					
Female	332 (38.8)					
N/A	189 (22.1)					
**Tumor primary site (n = 764)**	**No. (%)**	**Negative (%)**	**Positive (%)**	**No. fluor**	***P* value**	**Prop [95% CI]**
Total type	764	318 (45.3)	384 (54.7)	702		
Lung	337 (44.1)	130 (42.2)	178 (57.8)	308	<.01^a^	0.69 [0.53, 0.81]
Small cell lung cancer	8 (1.1)	3 (37.5)	5 (62.5)	8		
Nonsquamous cell carcinoma	251 (32.9)	109 (49.6)	111 (50.5)	220		
Squamous cell carcinoma	22 (2.9)	9 (50.0)	9 (50.0)	18		
N/A	56 (7.3)					
Breast	107 (14.0)	36 (36.7)	62 (63.3)	98	<.01^a^	0.65 [0.44, 0.82]
Gastrointestinal/colorectal	77 (10.1)	38 (51.4)	36 (48.7)	74	<.01^a^	0.58 [0.36, 0.78]
Urogenital	27 (3.5)	16 (61.5)	10 (38.5)	26	.54	0.39 [0.22, 0.58]
Melanoma	92 (12.0)	49 (59.8)	33 (40.2)	82	.11	0.46 [0.29, 0.63]
Renal cell	17 (2.2)	5 (33.3)	10 (66.7)	15	.63	0.66 [0.40, 0.85]
Squamous cell carcinoma (skin)	9 (1.2)	7 (77.8)	2 (22.2)	9	.12	0.34 [0.03, 0.91]
Other	84 (11.0)	32 (40.5)	47 (59.5)	79	<.01^a^	0.59 [0.31, 0.81]
Unknown	14 (1.8)	5 (45.5)	6 (54.6)	11	.39	0.55 [0.24, 0.82]
**Histology (n = 464)**	**No. (%)**	**Negative (%)**	**Positive (%)**			
Total type	464	262 (56.5)	202 (43.5)			
Adenocarcinoma	292 (62.9)	174 (59.6)	118 (40.4)			
Small cell carcinoma	18 (3.9)	7 (38.9)	11(61.1)			
Squamous cell carcinoma	23 (5.0)	13 (56.5)	10 (43.5)			
Tubular breast cancer	9 (1.9)	3 (33.3)	6 (66.7)			
Ductal breast cancer	15 (3.2)	2 (13.3)	13 (86.7)			
Clear cell carcinoma	26 (5.6)	16 (61.5)	10 (38.5)			
Malignant melanoma	23 (5.0)	16 (69.6)	7 (30.4)			
Other	58 (12.5)	31 (53.5)	27 (46.6)			

No. (%), number of (percentage); Prop, proportion using random-effects model.

a Significant.

Sex was reported in n = 855 patients. Primary tumor site was reported in n = 764 patients. Histology was reported was reported in n = 464 patients. Left column shows total number of patients; Right column shows those sites with reported negative and positive fluorescence.

**FIGURE 3.**
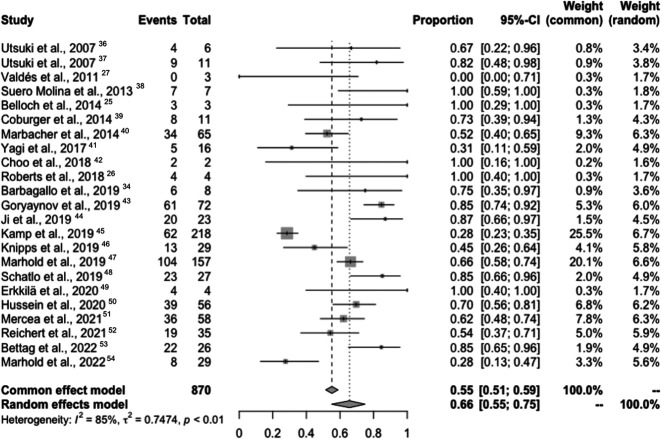
Pooled meta-analysis of overall prevalence of 5-ALA-PpIX fluorescence-positive metastatic lesions. 5-ALA, 5-aminolevulinic acid; PpIX, induced protoporphyrin IX.

**FIGURE 4.**
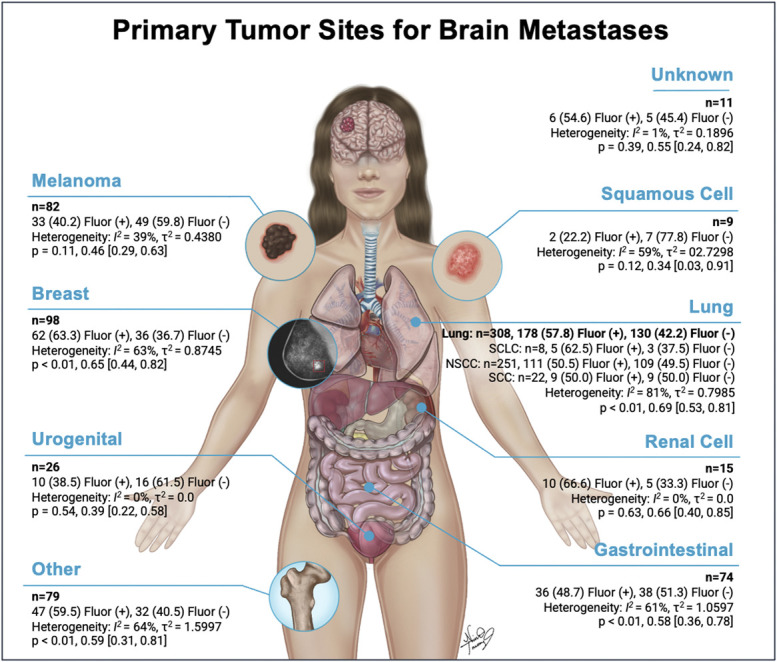
Fluorescence distribution based on primary tumor sites. Primary tumors sites for brain metastases with those showing negative (Fluor −) and positive (Fluor +) fluorescence. n = number (%).NSCC, nonsquamous cell carcinoma; SCC, squamous cell carcinoma; SCLC, small cell lung cancer. *© 2024 Nina Truong· All rights reserved*.

The pooled overall prevalence of fluorescence-positive metastatic lesions was 66% (95% CI 55%-75%; I^2^ = 85%, *P* < .01) (Figure 3), indicating that 66% of the population of 870 metastatic lesions are likely to be fluorescence positive, with a high degree of heterogeneity demonstrated by the I^2^ = 85% and with a 95% CI of 55%-75%. Specifically, the pooled overall prevalences of fluorescence-positive metastatic lesions based on the primary tumor site are shown in Figure 4 with further breakdown by primary tumor site in **Supplemental Digital Content 6** (http://links.lww.com/NS9/A38). Primary tumor sites that were statistically significantly (*P* < .01) more likely to display positive fluorescence were the lung (69%), breast (65%), gastrointestinal/colorectal (58%), and other (59%) (Table 3). The Egger regression for funnel plot asymmetry was significant showing a negative skewness with *P* < .01.

## DISCUSSION

5-ALA/PpIX has been shown to be useful and is currently approved as an adjunct for the visualization of malignant glioma tissue during surgery.^8^ However, despite BMs being the most common brain tumor, there is no FDA-approved FGS agent for use in this tumor population. The 57 articles found on 5-ALA/PpIX fluorescence in BMs represent the extent of the English literature on the topic describing the clinical experience of 5-ALA/PpIX FGS in BMs. This systematic review is the largest to date with 23 articles encompassing a total of 855 patients with 870 metastatic lesions. The patient population in this study is representative of the typical distribution of BMs with a male-to-female ratio of 1:1, and metastases percentages for the 3 most common primary tumor sites (ie, lung (44.1%), breast (14.0%), and melanoma (12.0%) also correspond to the expected likelihood of these metastases in the general population at 45%, 15%, and 10%, respectively.^24,55^

Our proportional meta-analysis using a random-effects model found the prevalence of fluorescence-positive metastatic lesions to be 66%, which is in line with the 2 single largest published series.^45,47^ Based on our proportional meta-analysis, the BMs primary tumor sites with a statistically significant pooled prevalence displaying fluorescence-positive lesions were the lung (69%), breast (65%), gastrointestinal/colorectal (58%), and other (59%). Adding to the variability of the data, a broad range of fluorescence positivity between the different primary cancer pathologies was observed. The broad range of different primary cancers and their BMs showing that positive fluorescence can be appreciated in individual studies with 10 or more patients with metastases, where the percentage of patients who showed visible fluorescence ranged from 27.6% in Marhold et al 2022 to 87% in Ji et al.^44,54^ For example, the low proportion of patients who displayed fluorescence in Marhold 2022 study is possibly secondary to all metastases representing melanomas, which have been shown to be less likely to display visible fluorescence when compared with other BMs, increasing the variability.^47,54^

Most studies described here used a conventional surgical microscope modified for fluorescence imaging. However, recent studies have shown using handheld optical spectroscopy probes for quantitative fluorescence,^58^ multipeak fluorescence,^20^ confocal microscopy,^59^ hyperspectral imaging technologies,^60,61^ or more recently with exoscopes, endoscopes, and fluorescence surgical loupes; visualization and detection of PpIX fluorescence can be significantly enhanced and thus, might enable more complete surgical resections.^25,42^ In a small cohort of BMs, Valdes et al^58^ showed that using a quantitative spectroscopic handheld probe, it was possible to improve the detection rates of metastatic tissue followed by observations by Choo et al^42^ (100%, n = 2/2) and Bettag et al^53^ (84.6%, n = 22/26) using endoscope-based systems. Similarly, Belloch et al^25^ reported detection in 100% of cases using an exoscope system. The Bettag study^53^ was able to observe fluorescence in 5 of 7 melanomas, which as noted above appear to be the least likely metastatic tumors to fluoresce using conventional surgical microscopes/filters. Other groups are exploring different excitation wavelengths for alternative and optimal fluorescence-guided outcomes.^26^ Thus, these findings suggest that a significant proportion of fluorescence-negative metastatic tumors, as assessed using conventional surgical microscopes, actually contain a diagnostically significant greater amount of PpIX compared with normal brain tissue. Thus, if we used/developed improved optical technologies/parameters, such “fluorescence-negative” tumors might be “fluorescence-positive.”

Such information could inform surgeons when making decisions regarding the use of 5-ALA/PpIX in BMs. For example, in cases of a primary with a high likelihood of showing positive fluorescence, such as renal cell, 5-ALA/PpIX may provide utility. However, in cases of a primary with a low likelihood of showing positive fluorescence, 5-ALA/PpIX may not be useful. Thus, our study can help surgeons make informed decisions regarding the use of 5-ALA/PpIX in BMs and could serve as a surgical adjunct in a high proportion of patients if we have a priori knowledge of their primary (eg, lung with 69% pooled prevalence for fluorescence-positive lesions). Furthermore, even if agnostic regarding primary cancer, 5-ALA/PpIX may be useful, given its pooled prevalence of 66%. In addition, given the greater total numbers of BMs in the general population compared with HGG, 5-ALA/PpIX in BMs might have a greater overall impact given the greater number of patients likely to be treated.

These findings using advanced technologies for FGS other than conventional surgical microscopes are not surprising, given that the observed fluorescence through a surgical microscope has multiple factors that can negatively affect the signal; fluorescence emissions are split between multiple detectors in microscopes (observer/s, camera), while in other exoscopes and/or loupes, all fluorescence is sent to a single detector. Furthermore, light must travel a longer distance which affects the signal detected by a factor of 1/r^2^, from tissue to the final detector on a surgical microscope (surgeon, cameras) as compared with handheld probes (directly in contact), endoscopes (almost in contact), or exoscopes and loupes where there could be a shorter distance for the light to travel. Finally, microscopes perform single bandpass/longpass detection of fluorescence unto the naked eye or unto a color camera and do not account for the confounding effects of tissue optical properties that affect the detected fluorescence, making all current assessments subjective, and not quantitative, or they do not account for the complex spectral changes in fluorescence enabled by novel spectroscopic systems or hyperspectral systems to perform separation of PpIX, its photoproducts, and autofluorescence, enabling more accurate and sensitive detection of PpIX. In summary, as is the case with HGG, improved surgical technologies have the potential to significantly improve the ability to detect 5-ALA/PpIX during the resection of BMs and thus, improve surgical outcomes including local recurrence rates by helping remove microscopic disease.

### Limitations

This study and 5-ALA as a technique have limitations in utility and applicability for guiding resection of BMs. First, this study was limited to English-language articles only. Second, it does not correlate the presence of fluorescence with clinical outcomes including overall or progression-free survival, or with malignant features on intraoperative histopathology. The cost of administering 5-ALA and the varying possibility of encountering no fluorescence present challenges in routine use within patient populations. Other limitations involve the lack of consistent reporting of normal peri-tumoral tissue fluorescence, EOR, and gross total resection (the basis for the lack of inclusion in our results). Moreover, the observed heterogeneous nature of fluorescence along with the potential for fluorescence in what appears to be normal peri-tumoral tissue with no evidence of invasion adds complexity to 5-ALA's utility. Moreover, the limited data using MRI as the modality to assess EOR likely do not adequately account for residual microscopic disease. A lack of consistent data on the presence of microscopic tumor remnants postresection limits a comprehensive understanding of this critical aspect. In hopes of providing a starting point for future studies to address this limitation, what data we could gather on normal peri-tumoral tissue fluorescence, EOR, and gross total resection can be found in our **Supplemental Digital Content 2** (http://links.lww.com/NS9/A34). The typically high I^2^ statistic in meta-analysis is not concerning as it is due to differences in study location and timing. Prediction intervals are the best way to incorporate uncertainty in meta-analysis comparisons.^56,57^ The Egger regression showed publication bias toward smaller studies with extreme effect sizes. Despite this bias, these initial findings pave the way for future investigations using larger sample sizes.

## CONCLUSION

The use of 5-ALA/PpIX in BMs has shown varying results as an adjunct to surgical resection in some metastases more than others, namely lung, breast, and gastrointestinal/colorectal. Here we provide the most comprehensive review of the literature on 5-ALA/PpIX in BMs, with exciting results showing that some BMs are likely to demonstrate fluorescence, informing the surgical community on its possible use for CNS disease. Furthermore, initial results also show that advances in surgical optical technology and more explicit grading systems display promise to significantly improve fluorescence detection in these tumors and peritumoral regions. In summary, the data shown here can inform neurosurgeons regarding future studies on FGS for BMs. This work can help inform the field of FGS to guide additional studies beyond HGG to additional pathologies such as BMs, which account for most brain tumors and which might have the most oncologic impact.

## SUPPLEMENTAL DIGITAL CONTENT

**Supplemental Digital Content 1**. **Table 1**. Nonmetastatic patient and fluorescence data. Fluorescence data of the patients included in the studies reported in this review were nonmetastatic. Fluorescence reported in simple (positive/negative) or (negative/weak/strong) fluorescence, locations of tumors are displayed by supratentorial, infratentorial, or unclassified and then by specific location, No. (%) = number of (percentage). **Supplemental Digital Content 2**. **Datasheet**. Supplemental datasheet with review and extraction information. **Supplemental Digital Content 3**. **Figure 1**. Fluorescence distribution in nonmetastatic tumors. Fluorescence data of the patients included in the studies reported in this review were non-metastatic. Total number of patients was n = 896 in (left) low-grade gliomas, (middle) high-grade gliomas, and (right) meningiomas. Fluorescence was reported as negative (blue, Fluor—Neg) or positive (dark pink, Fluor—Pos). **Supplemental Digital Content 4**. **Figure 2**. Distribution of brain metastases based on intracranial location, total number of patients or samples, and gender. (**a**) Detailed tumor location comparison. (Left) Shows supratentorial tumor and (right) shows infratentorial tumor locations, with locations not specified shown as unclassified. (**B**) Total number of patients and samples. (Left) Shows total number of patients and (right) shows total number of samples, both for metastatic and nonmetastatic instances. (**C**) Available gender. Total number of female, male, and gender not specified patients. n = number of; dotted vertical line separates left from right sections in (**A**) and (**B**). **Supplemental Digital Content 5**. **Figure 3**. Fluorescence distribution in brain metastases and by primary tumor site. (**A**) Brain Metastases. Total number of patients were n = 870 in which n = 599 had “simple” fluorescence and n = 271 had “detailed” fluorescence reported. (Left) Total number of patients (n = 870) with reported negative (blue) or positive fluorescence (dark pink). (Middle) Subset of patients (n = 599) with reported fluorescence only based on “simple” fluorescence (negative [blue] and positive [dark pink]). (Right) Subset of patients (n = 271) with reported fluorescence based on detailed fluorescence (negative [blue], weak [light pink], or strong [dark pink]). n = number of; dotted vertical lines separate left, middle, and right sections. Fluorescence distribution based on primary tumor site. (**B**) Primary tumor site. Total number of patients with reported primary tumor site (n = 691) with negative (blue) or positive fluorescence (dark pink) in the following primary tumor sites (left to right): lung, breast, gastrointestinal/colorectal, urogenital, melanoma, renal cell, other, squamous cell, unknown. **Supplemental Digital Content 6**. **Figure 4**. Primary tumor sites for brain metastases forest plots; top to bottom (**Left**) melanoma, breast, urogenital, other (**Right**) unknown, squamous cell (skin), lung, renal cell, gastrointestinal.
